# The molecular basis of lysine 48 ubiquitin chain synthesis by Ube2K

**DOI:** 10.1038/srep16793

**Published:** 2015-11-23

**Authors:** Adam J. Middleton, Catherine L. Day

**Affiliations:** 1 Department of Biochemistry, Otago School of Medical Sciences, University of Otago, Dunedin 9054, New Zealand

## Abstract

The post-translational modification of proteins by ubiquitin is central to the regulation of eukaryotic cells. Substrate-bound ubiquitin chains linked by lysine 11 and 48 target proteins to the proteasome for degradation and determine protein abundance in cells, while other ubiquitin chain linkages regulate protein interactions. The specificity of chain-linkage type is usually determined by ubiquitin-conjugating enzymes (E2s). The degradative E2, Ube2K, preferentially catalyses formation of Lys48-linked chains, but like most E2s, the molecular basis for chain formation is not well understood. Here we report the crystal structure of a Ube2K~ubiquitin conjugate and demonstrate that even though it is monomeric, Ube2K can synthesize Lys48-linked ubiquitin chains. Using site-directed mutagenesis and modelling, our studies reveal a molecular understanding of the catalytic complex and identify key features required for synthesis of degradative Lys48-linked chains. The position of the acceptor ubiquitin described here is likely conserved in other E2s that catalyse Lys48-linked ubiquitin chain synthesis.

Degradation by the 26S proteasome determines the abundance of cellular proteins, and regulates many critical processes such as cell cycle control, inflammation and apoptosis^1^,^2^. The susceptibility of proteins to degradation by the proteasome is largely driven by conjugation with ubiquitin^3^. Proteins can be modified by the addition of single ubiquitin moieties or polymeric chains^4^,^5^,^6^,^7^. Both chain length and the number of modified substrate lysine residues appear to determine the efficiency of protein degradation, with ubiquitin chains linked by lysine 48 (Lys48) serving as the most efficient trigger of degradation.

Assembly of ubiquitin polymers onto substrate proteins initially involves reaction between the C-terminal glycine of an E2-conjugated donor ubiquitin (Ub^D^) and the nitrogen of a substrate lysine. Once modified, lysine residues on the substrate-linked ubiquitin can react with Ub^D^ resulting in chain formation. It is generally accepted that the E3 ubiquitin ligases specify the substrate to be modified, while the ubiquitin-conjugating E2 enzymes position the acceptor ubiquitin (Ub^A^) and primarily dictate the chain linkage type^8^,^9^. However, many E3s accelerate catalysis, and thus the formation of polyubiquitin chains, by stabilising the E2-conjugated Ub^D^ in a ‘closed’ conformation where it contacts helix 2 (α2) of the E2^10^,^11^,^12^,^13^. In this conformation the tail of Ub^D^ is locked in position so that the thioester bond is primed for nucleophilic attack by the incoming lysine.

The machinery that builds ubiquitin chains of all linkages is highly conserved; however, the position and orientation of Ub^A^ is variable as it specifies the type of chain linkage. Notably, in the crystal structure of the heterodimeric Ube2N-Ube2V1 conjugate the non-catalytic Ube2V1 binds Ub^A^ so that Lys63 is positioned to attack the thioester linkage between Ube2N and Ub^D^^14^. In contrast, formation of a catalytically competent Ube2S, which builds Lys11-linked chains, appears to depend on electrostatic interactions between the surface surrounding Lys11 in Ub^A^ and the active site^15^. Additional contacts made by the E3 help position Ub^A^ so that Lys11-linked chains are specified^16^,^17^. A number of E2s have the capacity to build Lys48 chains but a unified mechanism has not been reported. For example, dimerization of Ube2G2 combined with additional E2- and ubiquitin-binding domains in its cognate E3, gp78, are required for Lys48 chain synthesis^18^,^19^. Whereas, Chong *et al.* demonstrated that the Tyr59-Glu51 loop on Ub^A^ constructs an ‘engaging zone’ that is critical for formation of Lys48 chains by Ube2R1 (Cdc34)^20^. Both Ube2G2 and Ube2R1 have sequence features not found in other E2s and it seems unlikely that they provide a paradigm for Lys48 chain synthesis.

The ubiquitin conjugating enzyme Ube2K (also known as E2-25 K and Huntington Interacting Protein 2) is a Class II E2 that preferentially synthesizes Lys48-linked chains on monoubiquitylated substrates, or forms Lys48-linked diubiquitin in the absence of an E3^21^. This Ubc1 homologue^22^ is highly induced in the brains of individuals with Alzheimer’s disease, and is upregulated in neuronal cells after exposure to the amyloid-β peptide^23^. In this scenario, it has been proposed that Ube2K may build chains with a faulty variant of ubiquitin (UBB + 1), leading to inhibition of the 26S proteasome^24^. Uniquely amongst E2 proteins, Ube2K has a C-terminal ubiquitin associating domain (UBA). The UBA domain is not required for the synthesis of Lys48-linked chains^25^, but may act to tether the E2 to ubiquitylated substrates thereby enhancing elongation of Lys48 chains^26^.

The preferential synthesis of Lys48 chains by Ube2K provides a good model system for understanding the molecular determinants of E2-catalysed Lys48 chain synthesis. Here we show that Ube2K is a monomeric E2 and the position of Ub^D^ is similar to that observed elsewhere, while several key contacts between Ube2K and Ub^A^ orient the incoming ubiquitin. A structural model of the catalytic complex that forms Lys48-linked ubiquitin chains reveals the molecular basis for specific chain elongation by Ube2K. This model highlights key features that will likely underpin Lys48 chain formation by other E2s.

## Results

### The crystal structure of a Ube2K~Ub conjugate

In order to ensure the formation of a stable isopeptide bond between Ube2K and ubiquitin, we mutated the active site Cys92 to Lys. To prevent the undesirable conjugation of ubiquitin to Lys97, which is in close proximity to the active site, Lys97 was mutated to Arg. This K97R mutation had no effect on the activity of Ube2K (Supplementary Fig. S1a), and as previously reported^21^ purified Ube2K efficiently promotes formation of diubiquitin (diUb) in the absence of an E3.

**Figure 1:**
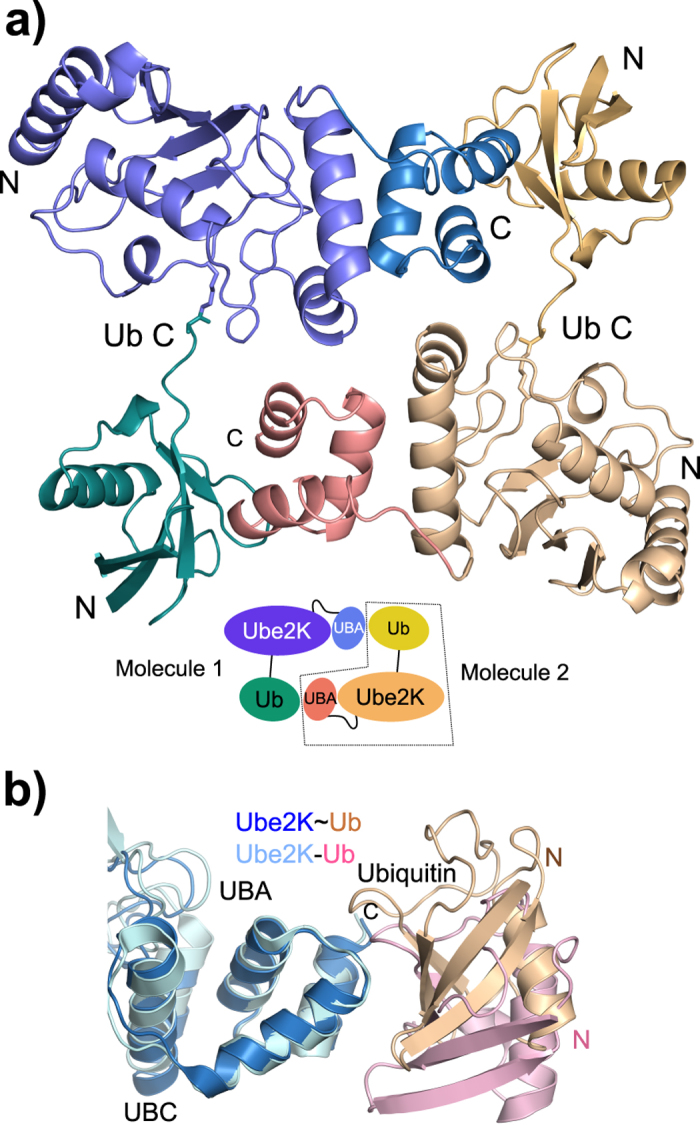
Crystal structure of Ube2K. (**a**) Cartoon representation of the crystal structure of the Ube2K~Ub conjugate and one of its symmetry mates highlighting the interaction between the molecules. Inset: schematic of the structure indicating the two molecules. (**b**) Overlay of the UBA domain of the Ube2K conjugate (blue and beige) and the Ube2K:Ub complex (pale blue and pink; PDB ID: 3K9P) showing the shift in the position of ubiquitin.

The isopeptide-linked Ube2K~Ub conjugate crystallized in spacegroup C2 with one molecule per asymmetric unit and data were processed to 2.1 Å (Table 1). In the structure, two conjugates associate with the ubiquitin moiety of one conjugate packed against the MGF-motif^27^ of the UBA domain of the neighbouring molecule (Fig. 1a). In this conformation ubiquitin is locked in an open position and there are minimal contacts between Ube2K and its conjugated ubiquitin.

An overlay of the UBC domain of the Ube2K conjugate structure with that of the previously reported Ube2K UBA:ubiquitin complex^24^ has an RMSD of 0.48 Å. In contrast, the position of both the UBA domain and ubiquitin in our structure is shifted (Fig. 1b). When the two UBA domains are aligned, even though the same residues mediate contacts between ubiquitin and the UBA domain, ubiquitin is rotated relative to the standard ubiquitin:UBA interaction (Supplementary Fig. S1b–d). Even though crystal contacts may help stabilize this conformation, this structure suggests a more plastic Ub-UBA interface than previously reported.

The apparent dimeric configuration of Ube2K~Ub did not suggest a catalytically competent complex had formed, as Lys48 of the UBA-associated ubiquitin is ~30 Å from the active site of its partner. Indeed, when analysed at 30 μM using MALS coupled to a size-exclusion column both the E2 and the conjugate behaved as monomers (Supplementary Fig. S1e). This is in agreement with recent work by Cook *et al.* who showed that the Ube2K conjugate was monomeric at concentrations up to 280 μM and calculated a dimerization constant of more than 1 mM^26^. Together, these data suggest that Ube2K can synthesize Lys48-linked chains as a monomeric E2.

### The donor ubiquitin interacts with α2 of Ube2K

Recently, several structures of Ube2D~Ub conjugates in complex with RING E3s have shown how the conjugated ubiquitin interacts with the E2^10^,^11^,^12^,^13^. Importantly, the Ile44 hydrophobic patch of Ub^D^ contacts the C-terminal portion of α2 in the E2 and these interactions are required for formation of the catalytically primed closed conformation^10^,^11^,^28^. In the NMR model of the thioester-linked Ubc1~Ub conjugate, similar contacts between ubiquitin and the E2 were reported^29^. To explore the role of this interaction in formation of Lys48-linked chains by Ube2K we mutated residues in α2 (L111A, L115A, A118L) and assessed the ability of the mutant proteins to synthesize ubiquitin chains in the absence of an E3 ligase. Quantification of the assays (Supplementary Fig. S2b) showed that formation of diUb was decreased for all these mutants (Supplementary Fig. S2b), with activity most severely reduced for the A118L mutant (Supplementary Fig. S2b,c). In the NMR model of Ubc1~Ub, the equivalent residue, Gln114, was reported to interact with Ub^D^ and the reduced activity seen here is consistent with this residue having a similar role^29^. Surprisingly, mutation of Glu121 in the adjacent α2-α3 loop resulted in an increase in diUb formation (Supplementary Fig. S2b,c) without showing any loss of specificity for formation of Lys48-linked chains (Supplementary Fig. S2d). The equivalent Asn in Ube2D2 interacts with the C-terminal tail of the donor ubiquitin^10^, and the observed increase in chain synthesis suggests that either the reactivity of the thioester linkage between Ube2K and Ub^D^ has been increased, or that the closed conformation has been stabilised.

Together, these data suggest that the donor ubiquitin likely adopts a closed conformation that depends upon interaction with α2 of Ube2K, and in agreement with analysis of other E2s, this interaction promotes ubiquitin transfer.

### Identification of the acceptor-binding site on Ube2K

**Figure 2:**
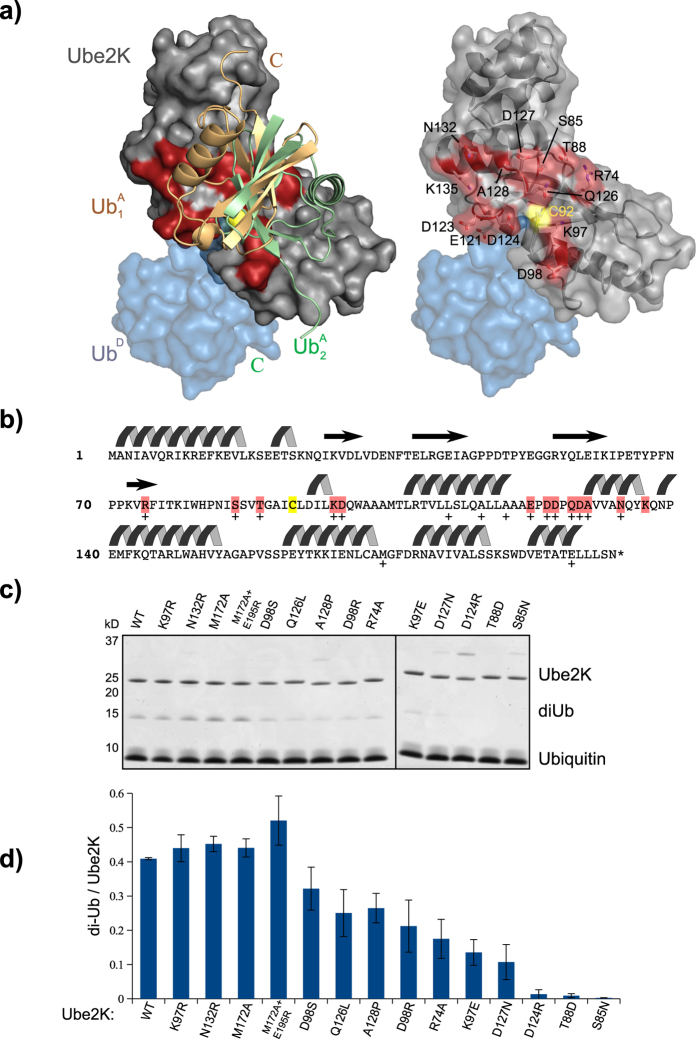
HADDOCK model of the Ub^A^-Ube2K~Ub complex. (**a**) The Ube2K~Ub conjugate is shown as a surface representation (grey and blue, respectively). Left: top two predictions of the position of Ub^A^ are shown as a ribbon in beige and light green. Residues predicted to interact with Ub^A^ are highlighted in red. On the right, the ubiquitin molecules are removed and residues predicted to bind Ub^A^ are indicated in black text. (**b**) Amino acid sequence and secondary structure of Ube2K. Residues within five Å of Ub^A^ in the model are highlighted in pink. Initial Ube2K mutations are indicated (+). (**c**) Coomassie-stained SDS-PAGE of a multi-turnover diUb assay performed with each Ube2K mutant quantified in panel (**d**). (**d**) Quantification of diUb formation. Bars represent the mean of triplicate measurements, error bars represent standard deviation.

How does a monomeric E2 build only Lys48-linked chains? In order to model Lys48 chain synthesis, we used HADDOCK^30^ to dock Ub^A^ to the Ube2K~Ub conjugate. As one input molecule, we generated a model of the Ube2K conjugate based on the structure of Ubc1~Ub in the closed conformation (PDB ID: 1FXT); while for the second molecule (Ub^A^), we used the crystal structure of ubiquitin. In order to ensure that Lys48 of the acceptor ubiquitin remained close to the active site cysteine of Ube2K, an unambiguous distance restraint was specified between the N_z_ atom of Lys48 of Ub^A^ and the S atom of Cys92 of Ube2K. The top two clusters generated by HADDOCK had similar energies and scores. In both clusters, the predicted Ube2K-Ub^A^ interface comprised an area surrounding the active site of Ube2K, and a surface adjacent to Lys48 on Ub^A^ (Fig. 2a). The primary discrepancy was with the orientation of the ubiquitin molecule, which was rotated ~180° in the two models as properly positioning Ub^A^ was not possible with the limited information provided. Despite this ambiguity, the initial docking results allowed mutations to be designed in Ube2K that would be predicted to disrupt Ube2K-Ub^A^ interactions (Fig. 2a,b).

Following purification, the ability of the Ube2K mutants to be charged with ubiquitin was assessed (Supplementary Fig. S3a). Ubiquitin in which all the lysine residues had been mutated to arginine (K_0_ ubiquitin) was used for this assay because it precluded chain formation and therefore Ube2K discharge. Mutants that could not be charged were not further investigated (Supplementary Fig. S3b). Next, the integrity of the active site of Ube2K was evaluated in an autoubiquitylation assay that included RNF12, a RING E3 ligase that we have shown to be active with Ube2K (Supplementary Fig. S3c). In addition to the A118L mutant, which disrupts positioning of Ub^D^, the D124R mutant did not support RNF12 autoubiquitylation suggesting that the thioester in this conjugate is not susceptible to attack (Supplementary Fig. S3d). Neither mutant was used in future analyses of acceptor binding and polyubiquitin chain formation.

We next assessed the ability of active mutants to form Lys48-linked diUb in the absence of an E3 ligase. Because Ube2K only forms Lys48-linked diUb^21^, we assumed that Ub^A^ must be specifically positioned and that disrupting binding of Ub^A^ would result in diminished diUb synthesis. As expected based on the HADDOCK model some of the mutations disrupted diUb formation. This was apparent in time course assays and when endpoints were quantified (Fig. 2c,d, Supplementary Fig. S4) Mutations targeted to off-site positions such as Met172 and Glu195, and some near the catalytic site resulted in no decrease in the ability for Ube2K to synthesize diUb. Together these data identify residues adjacent to the catalytic cysteine that are required for binding of Ub^A^ to Ube2K.

### Identification of the Ube2K binding surface on the acceptor ubiquitin (Ub^A^)

**Figure 3:**
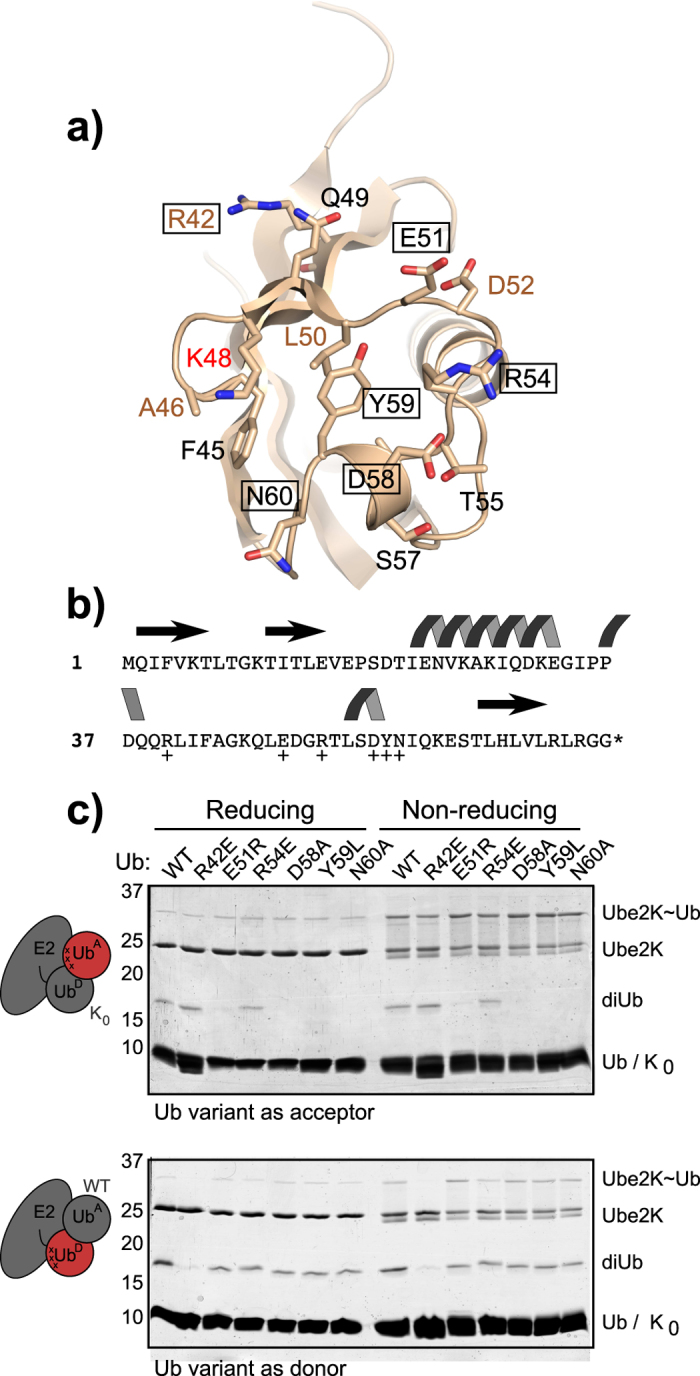
Role of the Lys48 face in ubiquitin chain synthesis. (**a**) Schematic showing the Lys48 face of ubiquitin with residues predicted to interact with the active site of Ube2K indicated in black. Beige-coloured text indicates residues on the surface that are not predicted to be involved in binding to Ube2K. Boxed residues have been mutated in this study. Carbon, nitrogen and oxygen atoms are coloured beige, blue and red, respectively. (**b**) Sequence of ubiquitin showing the elements of secondary structure, with mutated residues indicated (+). (**c**) Single turnover diUb assay (40 minutes) where the ubiquitin variant is either Ub^A^ (top) or Ub^D^ (bottom). The presence of diUb indicates the ubiquitin variant can support chain formation. Reducing and non-reducing samples are shown to evaluate charging.

In both of the top-scoring HADDOCK model clusters, a surface adjacent to Lys48 in Ub^A^ is predicted to interact with Ube2K (Fig. 3a,b). The importance of this region was evaluated using six ubiquitin mutants (R42E, E51R, R54E, D58A, Y59L and N60A). To determine if these mutations disrupted interaction of Ub^A^ with Ube2K, the E2 was first charged with K_0_ ubiquitin and then diUb formation was assessed following addition of each mutant Ub^A^. In this single-turnover assay, diUb synthesis was impeded for four of the mutants (E51R, D58A, Y59L, and N60A) suggesting that acceptor binding had been disrupted (Fig. 3c, top). To determine if any of the mutants, which failed to serve as Ub^A^, had altered the structure of ubiquitin we also evaluated their ability to be charged onto Ube2K and to act as Ub^D^ (Fig. 3c, bottom). Surprisingly, only R42E, which was active as Ub^A^, could not be charged. These data suggest that the ubiquitin surface centred on Lys48, which includes Asp58, Tyr59 and Asn60, is required for interaction of Ub^A^ with Ube2K.

### Orienting the acceptor ubiquitin with charge-swap mutants

**Figure 4:**
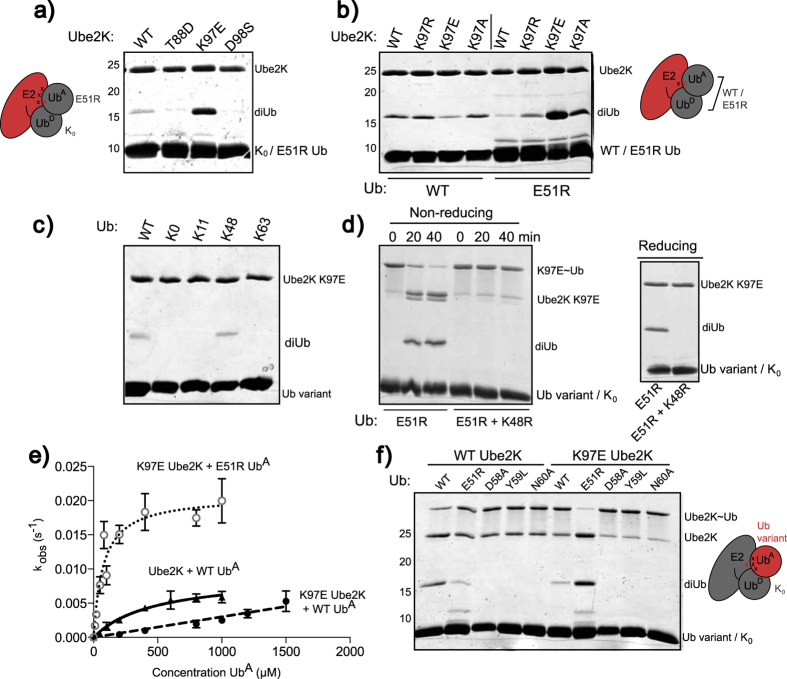
Probing the orientation of Ub^A^. (**a**) Single-turnover diUb assay where mutations that introduced a negative charge to the acceptor face of Ube2K were first charged with K_0_ for 30 min before being spiked with E51R ubiquitin as Ub^A^. The presence of diUb after 40 min incubation with Ub^A^ indicates successful recovery of activity. (**b**) Alternate amino acids were introduced at position 97 of Ube2K and tested with wild-type (lanes 1–4) and E51R ubiquitin (lanes 5–8) as Ub^A^ in a single-turnover diUb assay. (**c**) Single-turnover assays of K97E Ube2K spiked with single lysine variants of ubiquitin. DiUb only forms when Ube2K is mixed with wild-type and Lys48-containing ubiquitin as Ub^A^. (**d**) Single-turnover time course assay where K97E Ube2K was spiked with E51R or E51R/K48R ubiquitin as Ub^A^. Left: non-reducing time course. Right: reducing samples taken after forty-minute incubation with Ub^A^. (**e**) Initial rates of diUb synthesis were measured and plotted over a range of ubiquitin concentrations. Incubation time for wild-type Ube2K plus wild-type ubiquitin, and K97E Ube2K plus E51R ubiquitin was 40 s, while K97E Ube2K plus wild-type ubiquitin was 300 s. Data were fit to a non-linear curve by GraphPad Prism and apparent K_m_ and k_cat_ values were calculated (Supplementary Table S1). Error bars represent SEM. (**f**) Wild-type and K97E Ube2K tested with ubiquitin variants as Ub^A^ in a single-turnover diUb assay.

Having identified residues on Ube2K and Ub^A^ required for diUb synthesis, and presumably Ub^A^ binding, we sought to determine their relative orientation. To do this we utilised charge complementation experiments, which are based on the assumption that if two charged residues interact, switching the charge of either residue alone will disrupt binding and function. However, when both mutants are used in combination, the interaction and therefore activity will be recovered. Initially we used single-turnover assays that included conjugate prepared with three Ube2K mutants (T88D, K97E and D98S) and Ub^A^ that possessed the E51R mutation. Of the combinations tested, only the K97E form of Ube2K supported diUb synthesis when E51R was Ub^A^ (Fig. 4a). The high level of activity observed suggested that Lys97 in Ube2K and Glu51 in Ub^A^ might be in close proximity.

To further explore the role of Lys97, it was replaced with other amino acids and diUb synthesis with wild-type or E51R Ub^A^ was assessed (Fig. 4b). As before, substantial diUb chain synthesis only occurred when Lys97 was substituted by Glu, although replacement with uncharged Ala also supported some diUb synthesis (Fig. 4b). To determine whether the recovery of activity was due to a loss of specificity, K97E Ube2K was incubated with single lysine-containing ubiquitin variants. Of these, diUb could only be formed with wild-type and Lys48 ubiquitin, suggesting no loss of specificity (Fig. 4c). Furthermore, when Ube2K K97E was incubated with a double mutant form of ubiquitin in which Lys48 was mutated (E51R and K48R), diUb was not formed (Fig. 4d).

To assess whether Ube2K K97E disrupted Ub^A^ binding or altered catalysis, wild-type and mutant Ube2K were assayed in single-turnover assays with increasing amounts of ubiquitin or ubiquitin variants as Ub^A^ 31,32. The resulting diUb was quantified and the apparent k_obs_ (s^−1^) was determined as described previously^32^. In agreement with previously reported values, a baseline K_m_ of 476 μM and k_cat_ of 0.0091 s^−1^ were obtained for wild-type Ube2K and ubiquitin (Fig. 4e, Supplementary Fig. S5, Supplementary Table S1)^33^,^34^. In contrast, K97E Ube2K when incubated with wild-type Ub^A^ had a K_m_ that was larger than could be measured by this experiment. However, combining Ube2K K97E with Ub^A^ E51R resulted in complete recovery of both K_m_ and k_cat_ relative to the initial baseline level, suggesting that that these two residues interact directly (Fig. 4e, Supplementary Table S1). In fact, the K_m_ of Ub^A^ E51R for Ube2K K97E decreased by an order of magnitude, suggesting that the interaction with Ub^A^ is more favourable for this mutant combination.

Further assays were carried out to determine if any of the other ubiquitin mutants shown to eliminate Lys48 chain formation (Fig. 3c), including Y59L, which is known to disrupt Lys48 chain synthesis with Ube2K and Ubc1^20^,^32^, supported recovery of diUb synthesis with K97E Ube2K. As before, only the E51R Ub^A^-K97E Ube2K combination resulted in recovery of diUb formation (Fig. 4f). These experiments show that Glu51 of Ub^A^ contacts Lys97 in Ube2K.

### Docking the acceptor ubiquitin onto Ube2K

**Figure 5:**
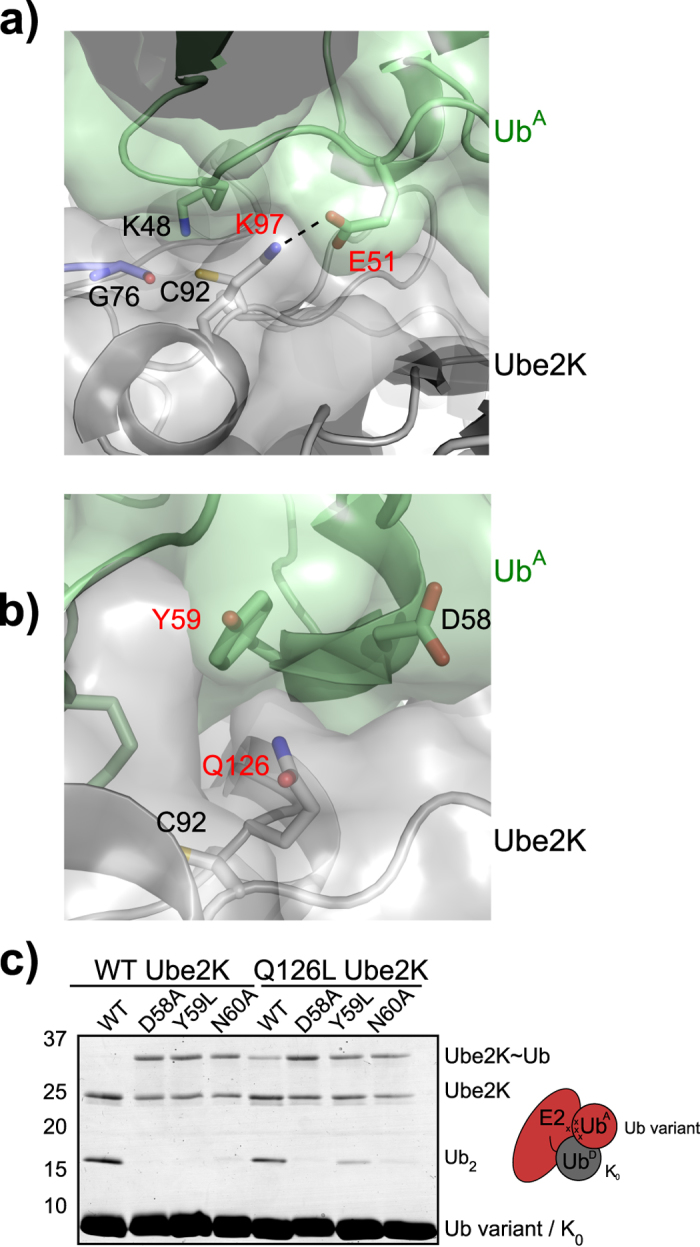
Evaluating the revised HADDOCK model. (**a**) Semi-transparent surface of the active site of the Ub^A^-Ube2K~Ub model showing the polar contact formed between Lys97 of Ube2K and Glu51 of Ub^A^. Ub^A^ is in pale green and Ube2K is in grey. Hydrogen bond indicated with a dashed line. (**b**) Schematic of the putative interaction between Gln126 of Ube2K and Tyr59 of Ub^A^. Colouring as in panel (**a**). (**c**) Non-reducing gel showing recovery of activity as a result of mixing Ube2K mutant Q126Y with Y59L ubiquitin as Ub^A^ in a 40 min single-turnover assay. See Supplementary Table S1 for apparent K_m_ and k_cat_.

Having established the interaction between Lys97 of Ube2K and Glu51 of Ub^A^ another HADDOCK docking run was carried out with this additional restraint included. The resulting models were grouped into only two clusters—as opposed to the six obtained in our initial dock. The first cluster contained the majority of the models (183/200) and had a lower HADDOCK score (meaning higher confidence in this cluster) and the buried surface area was larger. Furthermore, Ub^D^ in cluster 1 remained in the closed position whereas in the second cluster Ub^D^ had shifted so that it was no longer in contact with α2 of Ube2K. As expected the top model in the dominant group positioned Lys48 adjacent to the active site cysteine of Ube2K, while Glu51 interacted with Lys97 (Fig. 5a). Combining alternate lysines linkages with the Ube2K Lys97—Ub^A^ Glu51 interaction resulted in HADDOCK only satisfying one of the two restraints. This shows that Ub^A^ positioned so that Lys48 can attack the thioester is the only model that can satisfy the two experimental restraints.

The revised model of the catalytic complex also suggested that Gln126 in Ube2K was proximal to the aromatic group of Tyr59 in Ub^A^ (Fig. 5b). To evaluate this interaction, wild-type or Q126L Ube2K was incubated with D58A, Y59L and N60A ubiquitin. In this case, wild-type ubiquitin supported diUb synthesis by both forms of Ube2K, and as before all three mutants disrupted diUb synthesis by wild-type Ube2K (Fig. 5c). Kinetic assays with Q126L Ube2K and wild-type Ub^A^ resulted in the formation of diUb but the K_m_ was above the range we could calculate (Supplementary Table S1). Furthermore, for wild-type Ube2K and Y59L Ub^A^ no diUb was produced, precluding measurement of kinetic constants. However, endpoint assays clearly showed that the combination of Y59L Ub^A^ and Q126L Ube2K recovered activity (Fig. 5c) but the limited formation of diUb made it difficult to determine if rescue of activity was due to recovery of catalysis or binding (Supplementary Table S1). Previously, kinetic analysis of a Ubc1 mutant equivalent to Q126L Ube2K revealed a decreased k_cat_ but the K_m_ was unchanged^32^. The reason for the discrepancy is unclear but may be attributable to sequence differences distant from the active site of Ubc1 and Ube2K. Irrespective of the differences, the formation of diUb by the Y59L Ub^A^/Q126L Ube2K double mutant combination supports the model generated based on the K97E Ube2K and E51R Ub^A^ mutant pair.

### Serine 86 of Ube2K extends the β4- α2 loop and positions the acceptor ubiquitin

Sequence alignment reveals a single residue insertion in the β4-α2 loop preceding the active site Cys of Ube2K and its yeast homologue Ubc1, and as expected this loop is extended relative to other E2s, such as Ube2D2 (Supplementary Fig. S6a,b). The additional residue, Ser86, appears to stabilize the loop by hydrogen bonding to Asp127 (Supplementary Fig. S6b), and the model of the catalytic complex suggests small rearrangements occur upon Ub^A^ binding that result in contacts between Ser85 and Gln126.

**Figure 6:**
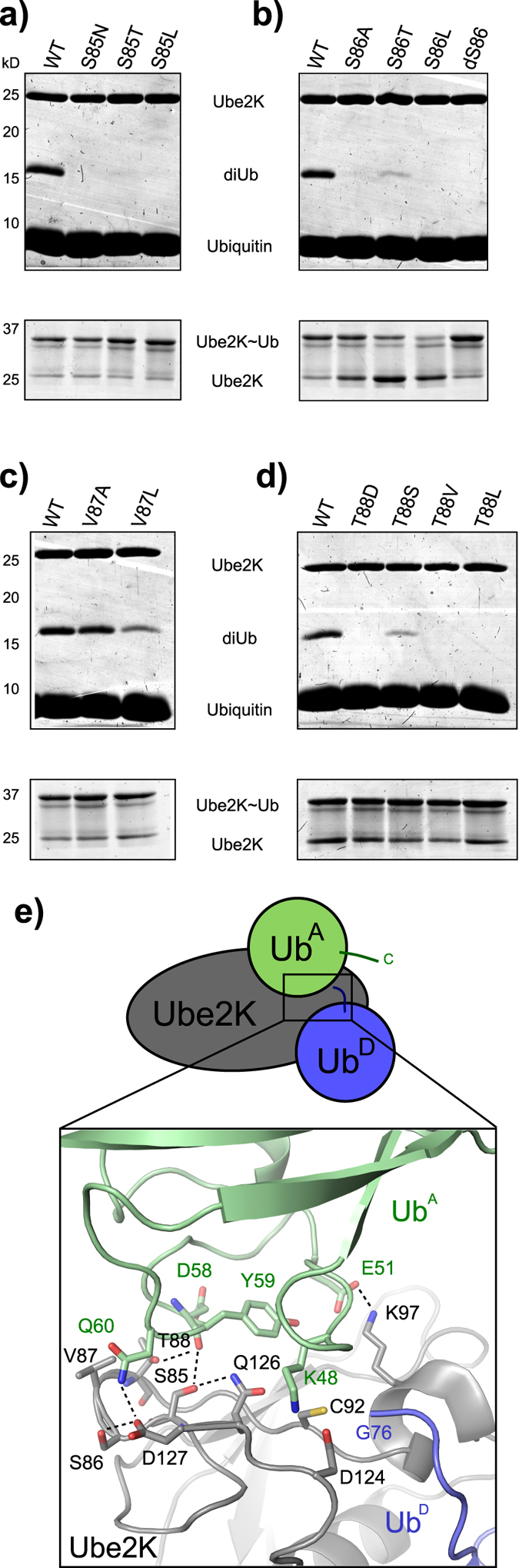
Residues 85–88 are critical for diUb synthesis. (**a**) Ube2K protein variants containing mutations targeting residue 85 were used in 40 minute diUb (upper; reducing gels) and charging reactions (lower; non-reducing gels). (**b–d**) DiUb and charging reactions as in panel A with Ube2K protein variants containing mutations to residues 86 (**b**), 87 (**c**), and 88 (**d**). (**e**) Schematic of the molecular model with a close up view of the predicted interaction between Ub^A^ and Ube2K~Ub. Interacting side-chains are shown as sticks and hydrogen bonds are indicated with dashed lines. Oxygen, nitrogen and sulphur atoms are red, blue and yellow, respectively. Carbon atoms are coloured as in the schematic above.

In order to probe the role of residues 85–88 in conferring Lys48 specificity, we mutated each residue. Mutation of Ser85 to a bulky Leu, or to the more similar Asn or Thr abolished diUb synthesis (Fig. 6a). This suggests that even the additional methyl group of Thr disrupts proper positioning of Ub^A^. We next mutated Ser86 to Ala, Thr, Leu and deleted the residue (delta-S86). Both S86A and delta-S86 resulted in a complete loss of activity, while S86T produced a small amount of diUb (Fig. 6b). S86L did not fully charge, so it was not closely investigated (Fig. 6b). Continuing our investigations into the importance of this loop, we found V87A retained wild type activity, while V87L had reduced activity, likely due to steric hindrance of the incoming Ub^A^ (Fig. 6c). Our anchored model predicts Thr88 makes a hydrogen bond to the main chain oxygen of Asp58 of Ub^A^. Mutation of Thr88 to Val or Leu abolished activity (Fig. 6d) while T88S showed some diUb synthesis. Thus, only when the hydroxyl group of Ser86 or Thr88 is retained is diUb formed, while all of the mutations made to position 85 result in a complete loss of diUb synthesis. Together, these data suggest that this stretch of residues has an essential role in specifying interaction of Ub^A^ with Ube2K.

In the model of Ub^A^-Ube2K~Ub catalytic complex, the side chains of residues 85 and 86 in Ube2K appear to stabilize the loop, and this rigidity is likely critical for selecting a particular Ub^A^ interface (Fig. 6e). Thr88 is predicted to make a hydrogen bond to Ub^A^ and Val87 also points towards Ub^A^. On the incoming Ub^A^, residues 58–60 are essential, and in the model they are positioned adjacent to the β4-α2 loop of Ube2K, and likely specify interaction between the active site of Ube2K and the Lys48-containing surface of Ub^A^.

## Discussion

The synthesis of linkage-specific ubiquitin chains requires that the incoming Ub^A^ is precisely oriented so that only the desired lysine can access the thioester bond that links the E2 to the Ub^D^ molecule. Here we show that the Lys48 specific E2, Ube2K, is monomeric and uses neither the UBA domain or another E2 molecule to position Ub^A^. Instead, residues adjacent to the active site on the E2 bind both the acceptor and donor ubiquitin molecules, positioning the incoming Ub^A^ so that only Lys48-linked chains are built. Ube2K is the only Lys48-specific E2 where chain formation does not depend on an acidic insertion^35^ or dimerisation^18^,^36^,^37^ of the E2, and therefore provides a template for understanding the minimal determinants of Lys48 chain formation.

Ubiquitin transfer by E2 ubiquitin conjugating enzymes, whether it be to a lysine residue in a substrate or another ubiquitin moiety, relies on stabilisation of an E2~Ub^D^ conjugate in the closed conformation. For most E2s the closed conformation of Ub^D^ depends upon interaction with an E3 ligase, which means that catalysis is tightly regulated. However, a few E2s like Ube2N (Ubc13), Ube2R1 (Cdc34) and Ube2K can synthesize ubiquitin chains in the absence of an E3^21^,^37^,^38^ suggesting that the ‘closed’ conformation of Ub^D^ is favoured by these E2s. Indeed, with no E3 present, the closed conformation of Ube2N is more frequently populated compared to that of Ube2D2^39^. Likewise, without requiring an E3 the Ubc1~Ub conjugate adopts a closed conformation^29^. For Ube2D, the E3-stabilized closed conformation relies on contacts between a hydrophobic face of Ub^D^ centred on Ile44 and α2 of the E2^10^,^15^,^39^,^40^. Similar catalytically primed Ub^D^ – E2 interfaces have been proposed for Ube2R1, Ube2G and Ube2S^15^,^34^,^41^, suggesting this interaction is conserved in all E2s. Indeed, mutation of residues in α2 of Ube2K disrupts chains synthesis (Supplementary Fig. S2).

As well as requiring proper positioning of Ub^D^, assembly of polyubiquitin chains depends on interaction of the E2 with Ub^A^. If the E2 synthesizes chains of only one linkage, as Ube2K does, then Ub^A^ must be precisely oriented so that only one lysine can access the active site. Previous attempts to measure Ub^A^ binding to an E2 by NMR have been unsuccessful^15^, likely due to the transient and low-affinity nature of this interaction. We therefore used mutagenesis combined with HADDOCK to build a model of the Ube2K catalytic complex. This model suggests that Lys48 chain synthesis by Ube2K is selected for by communication between the Glu51-Asn60 loop of Ub^A^ and residues around the active site of Ube2K. In particular, Lys97 of Ube2K contacts Glu51 of Ub^A^ (Fig. 5a), while the essential Ser85-Thr88 loop of Ube2K positions the other end of the Ub^A^ loop (Fig. 6). In this orientation, Lys48 of Ub^A^ is shielded from solution by Asp124, which is spatially identical to Asp127 of Ube2I^31^, and points towards the catalytic cysteine of Ube2K. As with many other E2s^15^,^31^, this Asp is indispensable for catalysis and mutating it results in an E2 that cannot efficiently ubiquitylate substrates at physiological pH (Supplementary Fig. S3d). Further stabilization of Ub^A^ in the catalytically competent complex is likely provided by interactions with the C-terminal tail of Ub^D^.

**Figure 7:**
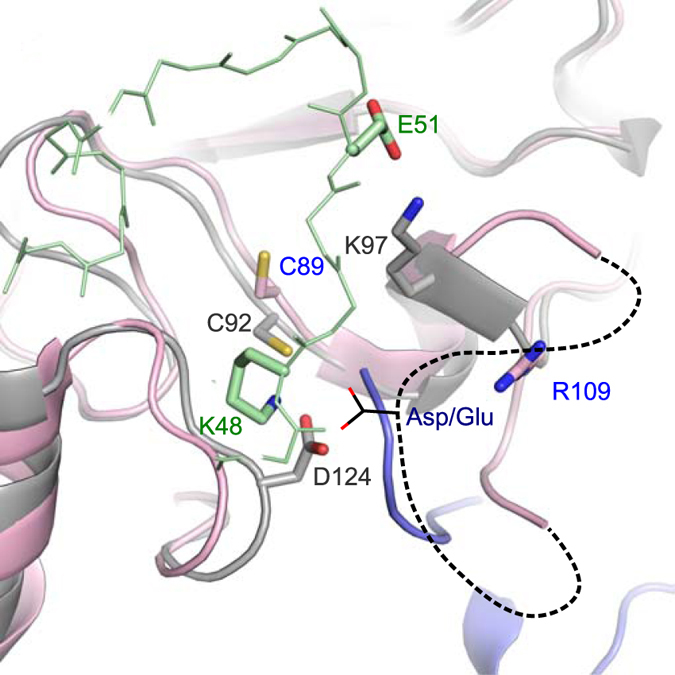
Structural model of Ub^A^ on Ube2G2. Overlay of Ube2K (grey) and Ube2G2 (pink; PDB ID: 4LAD) in the catalytic complex with Ub^A^ (green) and Ub^D^ (blue). Potential arrangement of the acidic β4α2 insertion in Ube2G is indicated by a dashed line, while the acidic group in this loop is drawn in black.

In our model, specificity of attack by Lys48 appears to be due to selection of one particular orientation of Ub^A^. Recent characterization of two other exclusively Lys48-synthesizing E2s, Ube2R1 and Ube2G2, has shown that a flexible acidic insertion adjacent to the active site is essential for catalysis^35^. In the case of Ube2R1, the same critical Lys48 surface of Ub^A^ as we have identified here was referred to as the ‘Cdc34-engaging zone'^20^. The acidic loop is flexible and missing from some structures but overlay of the UBC domain from Ube2R1 (PDB ID: 2OB4) with Ube2K in our model positions the acidic insertion close to the ‘Cdc34-engaging zone’ of Ub^A^. Similarly, overlay of Ube2G2 (PDB ID: 4LAD) with Ube2K positions Arg109 of Ube2G2, which was shown to communicate with Glu51 of Ub^A^, in a comparable position as Lys97 of Ube2K Glu (Fig. 7)^18^. In the overlay Arg109 and Glu51 are not in direct contact, but generation of electron density maps for 4LAD shows that Arg109 has weak density, and it is therefore likely to be flexible and rotamers that contact Glu51 are possible. This suggests the orientation of Ub^A^ in the Ube2K complex presented here is applicable to Ube2G2. Interestingly, Ube2R1/2 and Ube2G1/2 lack an acidic group in the position occupied by Asp124 in Ube2K (Supplementary Fig. S6a). For these Lys48-specific E2s it seems likely that the de-solvating group is provided by one of the conserved Asp or Glu residues in the essential acidic insertion^37^,^42^. Consistent with this, in the overlay with our model, the flexible β4α2 insertion is positioned such that one or more of the acidic residues is capable of interacting with Lys48 of Ub^A^ (Fig. 7). A conserved Glu residue in the insertion has also been shown to interact with the cognate E3 ligase of Ube2G2^40^, suggesting that the acidic loop serves multiple functions: selecting for a particular orientation of Ub^A^, binding the cognate E3 ligase, and de-solvating the incoming lysine.

Many E2s synthesize ubiquitin chains with several different linkages^8^ and therefore must bind Ub^A^ in more than one conformation. To enable synthesis of Lys48-linked chains a Lys or Arg at a position analogous to that occupied by Lys97 in Ube2K is required to contact Glu51 of Ub^A^ (Supplementary Fig. S6a). Lys or Arg residues are present at this position in the promiscuous Ube2D (Arg90 in Ube2D2) and Ube2E (Lys144 in Ube2E2) families (Supplementary Fig. S6) and consistent with this they both synthesize Lys48 chains^8^. Reflecting the importance of this residue in coordinating Ub^A^, mutating Arg90 of Ube2D2 to Glu (but not Ala) disrupts ubiquitin chain formation^43^.

Untangling the ubiquitin code requires a thorough understanding of the precise positioning of both Ub^D^ and Ub^A^ on each E2. Although the position adopted by Ub^D^ appears to be conserved for a number of E2s, chain specificity is directed by the position of Ub^A^, and in general this is not well understood. However, the E2 seems to be the ‘decider’ of chain specificity and the work performed here provides a more detailed understanding of how Lys48-specific E2s can position Ub^A^ for chain extension. Importantly, our model of the Lys48-specific catalytic complex reveals how monomeric E2s can build a specific polyubiquitin chain that is likely recapitulated by more promiscuous E2s.

## Materials and Methods

### Cloning and mutagenesis

The cDNA encoding Ube2K was purchased from Integrated Science and cloned with no tag into pET-21d, while the cDNA for ubiquitin was cloned into pET-3a. Ube2K and ubiquitin mutants were made using single-step site-directed mutagenesis^44^ and mutations were confirmed by sequencing (Genetic Analysis Services, University of Otago, Dunedin, NZ). The coding sequence for human E1 was purchased from AddGene. E1 was cloned into pET24b to encode a C-terminal His x6 tag. DNA encoding residues 530–624 of RNF12 was cloned into pGEX-6P3 with BamHI and EcoRI.

### Protein expression and purification

Wild-type and mutant Ube2K were transformed into BL21 *Escherichia coli* cells and grown at 37 °C in 50 mL of lysogeny broth supplemented with 50 μg/mL ampicillin or carbenicillin, 1 mM MgSO_4_, 1 × 5052 and 1 × NPS. Once cells had reached suitable density (OD_600_ ~ 2.0) they were transferred to 18 °C for overnight expression. Cells were spun at 3000 × g for 20 min and the supernatant was discarded. The pellet was resuspended in 4 mL of 50 mM MES-OH pH 6.0 and 1 mM EDTA. After sonication, cell debris was spun down at 15 000 × g and the supernatant was injected over a 5 mL Hi-Trap SP column. A 10 column volume linear salt gradient (0–1 M NaCl) was used to elute Ube2K. Peak fractions were diluted three-fold in 20 mM Tris-HCl pH 7.5 and injected onto a 5 mL Hi-Trap Q column. Bound protein was eluted with 500 mM NaCl at a final concentration of 1–3 mg/mL. Wild-type and mutant ubiquitin was expressed and purified as described elsewhere^45^. E1 and RNF12 were expressed and purified as described elsewhere^46^,^47^.

### Conjugation of Ube2K

In order to make a stable isopeptide-linked conjugate, the active site cysteine of Ube2K was mutated to lysine. An additional Lys near the active site (Lys97) was mutated to Arg. The modified Ube2K was charged with ubiquitin as described elsewhere^48^. The conjugate was purified on a 26/600 S75 column and peak fractions were pooled and desalted before being injected onto a 5 mL HiTrap Q column. Ube2K~Ub was eluted with 20 mM Tris-HCl pH 7.5, 500 mM NaCl at ~5 mg/mL. The fractions were pooled and immediately used to set up the PACT and JCSG crystallization screens (Molecular Dimensions).

### Crystallization and structure determination of Ube2K~Ub

Ube2K~Ub crystals initially grew in condition A3 of the JCSG + screen (0.2 M di-ammonium citrate pH 5.0, 20% PEG 3350). Optimization of the pH, PEG concentration, and microseeding resulted in diffraction-quality crystals with dimensions of approximately 0.1 × 0.05 × 0.02 mm. These were cryoprotected in the mother liqueur solution supplemented with 30% glycerol before being flash frozen in liquid nitrogen. Data with resolution to 2.1 Å were collected at 113 K at the Australian Synchrotron beamline MX1^49^. The dataset was processed with XDS^50^ and merged with Aimless^51^. A clear density map was obtained using PhaserMR with 1UBQ (ubiquitin) and 1YLA (Ube2K) as search models. Manual building was performed in Coot to place the terminal residues of ubiquitin^52^. Refinement was performed using Phenix Refine^53^ and PDBRedo^54^.

MALS (Dawn 8+, Wyatt) coupled to a Superdex S75 10/300 GL (GE) was used to determine the molecular mass of Ube2K and Ube2K~Ub at a concentration of 30 μM.

### Ube2K conjugate modelling and acceptor ubiquitin docking

A molecular model of the Ube2K conjugate was constructed using a structural alignment of the UBC of Ube2K (1YLA) with the NMR model of Ubc1~Ub (1FXT). The Cα atoms of the UBC domains were aligned in PyMol and the C-terminal glycine of ubiquitin was fused to the active site cysteine of Ube2K with a thioester bond. Acceptor ubiquitin docking was performed with the conjugate model (all chains) and ubiquitin (1UBQ) using the HADDOCK webserver^30^. For the docking run, unambiguous restraints were specified between Lys48 of ubiquitin and Cys92 of Ube2K; and between the C-terminal Gly of the donor ubiquitin and Cys92 of Ube2K. Passive residues were solvent accessible within 6.5 Å of the active site and Lys48 on Ube2K and ubiquitin, respectively. The C terminus of the conjugate model was indicated as not being negatively charged.

For the subsequent run, the Glu51:Lys97 interaction was specified as an unambiguous interaction in addition to the Lys48:Cys92 and Gly76:Cys92. Otherwise the docking was done identically to the original run.

### Ubiquitylation assays

For the charging assays, 0.1 μM E1, 4 μM E2 and 25 μM of K_0_ ubiquitin were incubated with 20 mM Tris-HCl pH 7.5, 50 mM NaCl, 2 mM MgCl_2_, 5 mM ATP, and 0.5 mM tris(2-carboxyethyl)phosphine at 37 °C for 40 min. Reactions were terminated by mixing with sodium dodecyl sulfate (SDS) loading dye with no reducing agent. For the assays with RNF12, E2s were charged as above and were then spiked with 5 μM RNF12 and incubated at 37 °C for 20 min. Reactions were terminated with SDS dye containing 2-mercaptoethanol. Multi-turnover diUb assays were performed as for the charging assays, but with wild-type ubiquitin and the samples were mixed with reducing SDS dye. The diUb and E2 were quantified with Image Studio Lite v4.0 (LI-COR Biosciences). The quantified diUb was divided by the amount of E2 as an internal control (diUb/Ube2K). A standard curve was generated over the range of diUb quantified in this study to ensure it had a linear response (Supplementary Fig. S2a).

For assaying ubiquitin mutants as acceptors, Ube2K was first charged with K_0_ for 30 min, then the reaction was spiked with 50 mM EDTA to terminate ubiquitin activation. After a two minute incubation on ice, ubiquitin mutants were added (Ub^A^) at a final concentration of 10–25 μM and the reactions were incubated at 37 °C for 40 min. For assessing ubiquitin variants as donors, Ube2K was charged with the ubiquitin mutants (Ub^D^) for 30 min before being quenched with EDTA and spiked with wild-type ubiquitin (Ub^A^). All reactions were terminated with SDS loading dye with or without reducing agent.

For kinetics assays, Ube2K or Ube2K variants were charged with K_0_ ubiquitin and quenched with EDTA as described above. The charged Ube2K was then spiked with wild-type or variants of ubiquitin as Ub^A^ at a final concentration of 10–1800 μM and incubated at 25 °C. Reactions were terminated by addition of non-reducing sample buffer. The apparent rate of diUb synthesis was measured by dividing the quantified diUb by the initial amount of charged Ube2K and then by the incubation time in seconds. This apparent k_obs_ (s^−1^) was plotted against Ub^A^ concentration and apparent K_m_ and k_cat_ were determined using Michaelis-Menten equations and GraphPad Prism. Incubation times with Ub^A^ before quenching with loading dye are indicated in Supplementary Table S1. Reactions were performed in triplicate.

## Additional Information

**How to cite this article**: Middleton, A. J. and Day, C. L. The molecular basis of lysine 48 ubiquitin chain synthesis by Ube2K. *Sci. Rep.* **5**, 16793; doi: 10.1038/srep16793 (2015).

## Supplementary Material

Supplementary Information

## Figures and Tables

**Table 1:** Summary of data collection and refinement statistics.

	Ube2K~Ub
Data collection	
Wavelength (Å)	0.9537
Resolution (Å)	47–2.1 (2.16–2.1)^a^
Space group	C2
Unit-cell parameters	
a, b, c (Å)	146.3 37.56 61
α, β, γ (^o^)	90 90.43 90
Total reflections	146692 (11885)
Unique reflections	19503 (1552)
Completeness (%)	99.2 (98.3)
I/σ(I)	10.8 (1.5)
R_merge_ (%)	16.2 (157.5)
CC^1/2^	0.997 (0.617)
Redundancy	7.5 (7.7)
Refinement	
R_work_ (%)	19.5
R_free_ (%)	23.5
Non-hydrogen atoms	
All atoms	2249
Protein	2174
Water	57
Heterogeneous atoms	18
Average B factors (Å^2^)	24.3
Ramachandran plot (%)	
Most favored regions	97.1
Allowed regions	2.2
Disallowed regions	0.7

^a^High resolution shell is shown in parentheses.
