# Supplementary material for: The molecular basis of lysine 48 ubiquitin chain synthesis by Ube2K
Source: Sci Rep. 2015 Nov 23;5:16793. doi: 10.1038/srep16793 (PMC4655369; doi:10.1038/srep16793)
Supplement: Supplementary Information [file srep16793-s1.pdf]

Supplementary Information

The molecular basis of lysine 48 ubiquitin chain synthesis by Ube2K

Adam J. Middleton, Catherine L. Day*

Department of Biochemistry, Otago School of Medical Sciences, University of Otago, Dunedin 9054, New Zealand

* Email: catherine.day@otago.ac.nz

Supplementary 1

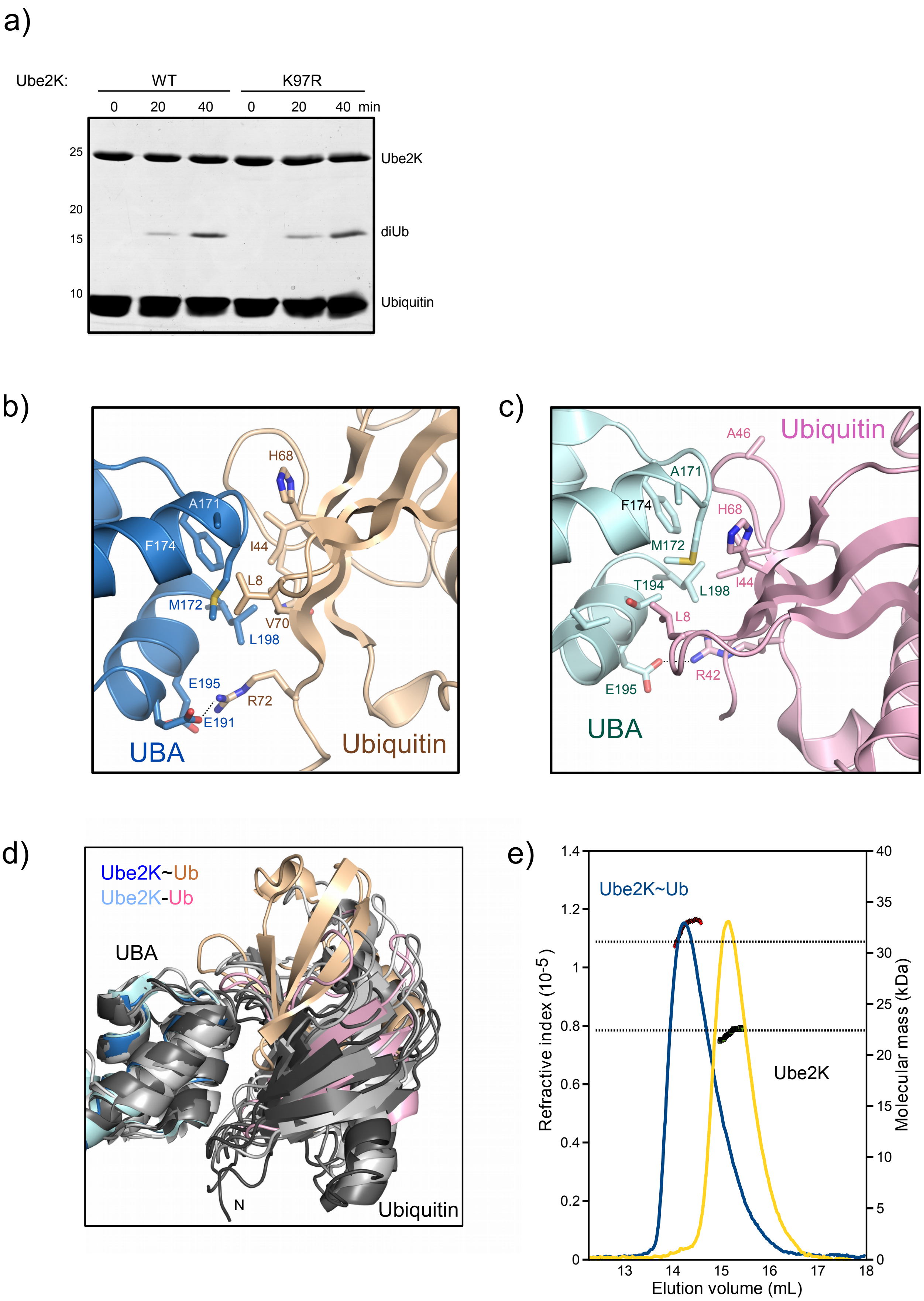

Supplementary Figure S1. Analysis of the Ube2K~Ub conjugate.

(a) Multi-turnover diUb time-course assay comparing wild-type and K97R Ube2K. **(b and c)** Close up schematic of the UBA:ubiquitin interaction seen in the **(b)** Ube2K crystal structure, and **(c)** Ube2K-ubiquitin complex (PDB ID: 3K9P). Shown in sticks are interacting side chains between Ube2K and ubiquitin. Oxygen, nitrogen and sulphur atoms are red, blue, and yellow, respectively. **(d)** Overlay of seven UBA:ubiquitin structures available on the PDB (PDB ID: 1WR1, 2MJ5, 2G3Q, 2MRO, 4UN2, TJY6, 3K9P) against the ubiquitin:UBA interface observed in the Ube2K~Ub conjugate crystal structure. Ube2K~Ub conjugate and UBA:ubiquitin structure (3K9P) are coloured as indicated. **(e)** MALS analysis of Ube2K and Ube2K~Ub at 30 µM shows that they are monomeric in solution. Calculated molecular mass of Ube2K (22.4 kDa) and Ube2K~Ub (31.0 kDa) are shown as dashed lines, while measured mass is represented as green (Ube2K) and red (Ube2K~Ub) squares.

Supplementary S2

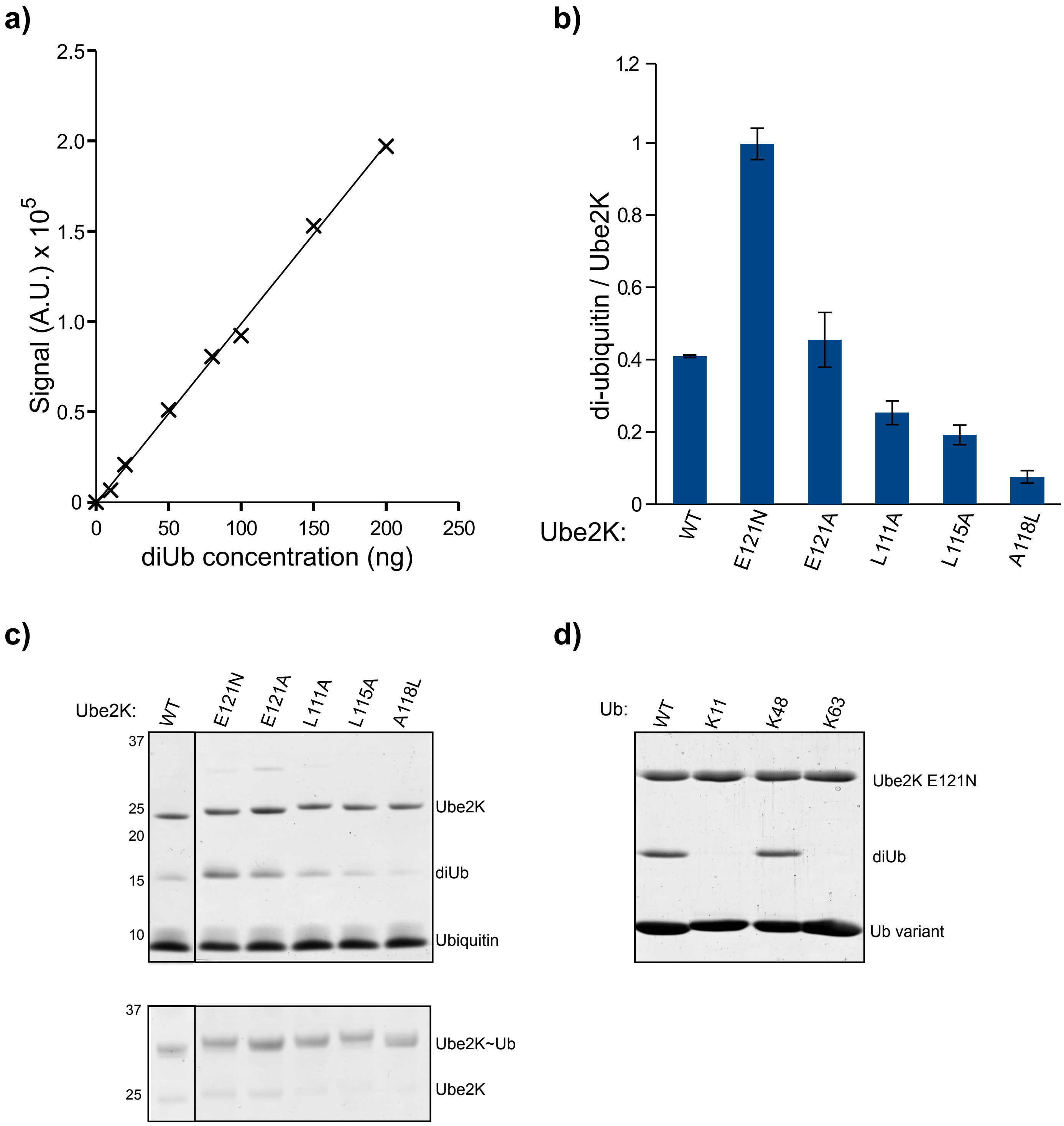

Supplementary Figure S2. Mutations targeting α2 of Ube2K disrupt ubiquitylation.

(a) Standard curve of quantified diUb from Coomassie-stained SDS PAGE gels. This standard curve was generated from diUb within the range used in this study ($R^2 = 0.99$). **(b)** Ube2K variants were assayed in a multi-turnover diUb assay and quantified in triplicate. Vertical bars represent standard deviation. **(c)** SDS PAGE gels of diUb (top) and charging assays (bottom) of Ube2K variants. Lane 1 is from Fig 2 (samples on same gel). **(d)** Single-turnover diUb assay performed with Ube2K mutant E121N combined with single-lysine containing variants of ubiquitin as Ub^A to test chain specificity.

Supplementary S3

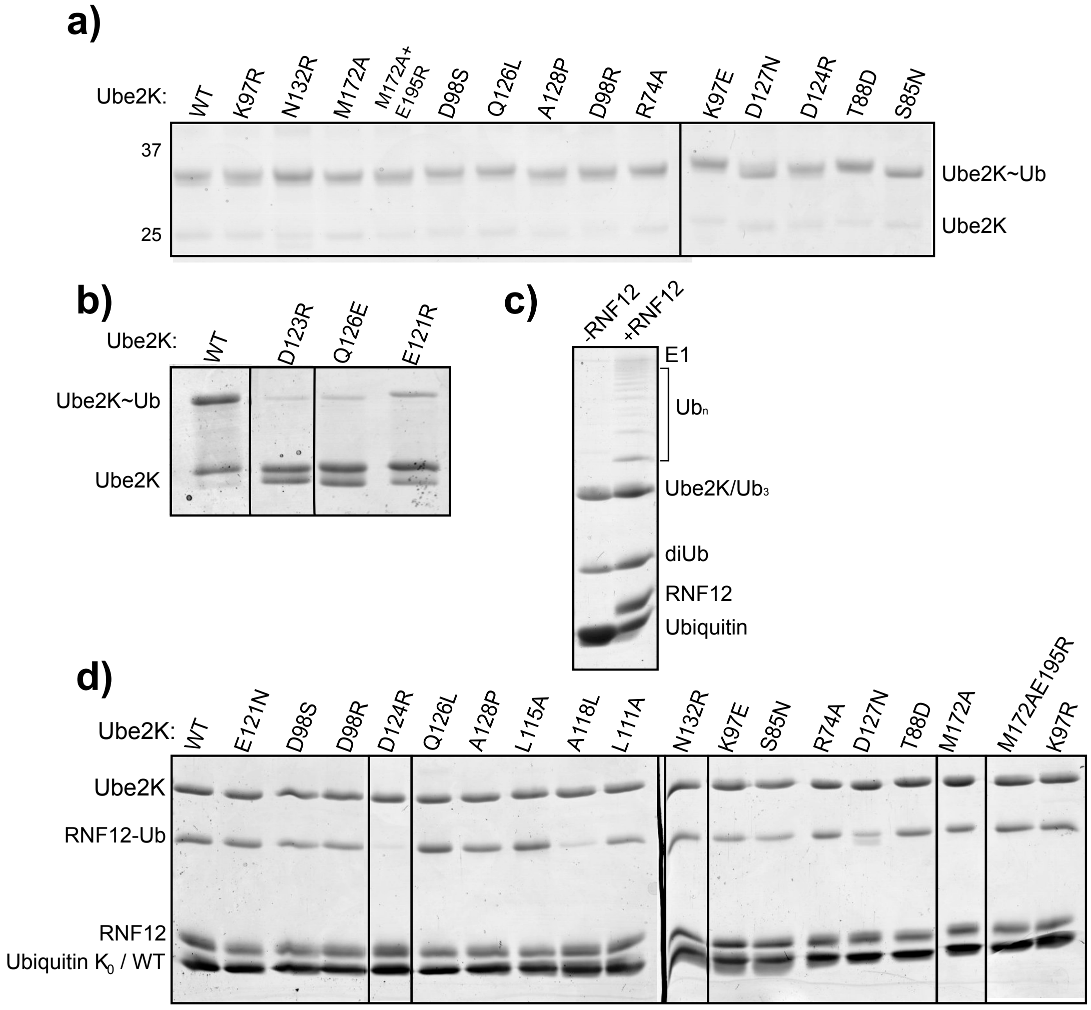

Supplementary Figure S3. Testing catalytic ability of Ube2K mutants.

(a) Charging assay showing that Ube2K variants analysed for diUb synthesis are properly folded and can interact with the E1 enzyme. **(b)** Assay of Ube2K variants that were found to be defective at charging with K_0 ubiquitin. These mutants were not further investigated in this study. **(c)** Multi-turnover ubiquitylation assay (60 min) with wild-type Ube2K in the presence and absence of RNF12. **(d)** Charged Ube2K mutants were spiked with 5 µM RNF12 to check the integrity of their active site by monitoring formation of monoubiquitylated RNF12. Only Ube2K variants D124R and A118L resulted in no production of ubiquitylated RNF12.

Supplementary S4

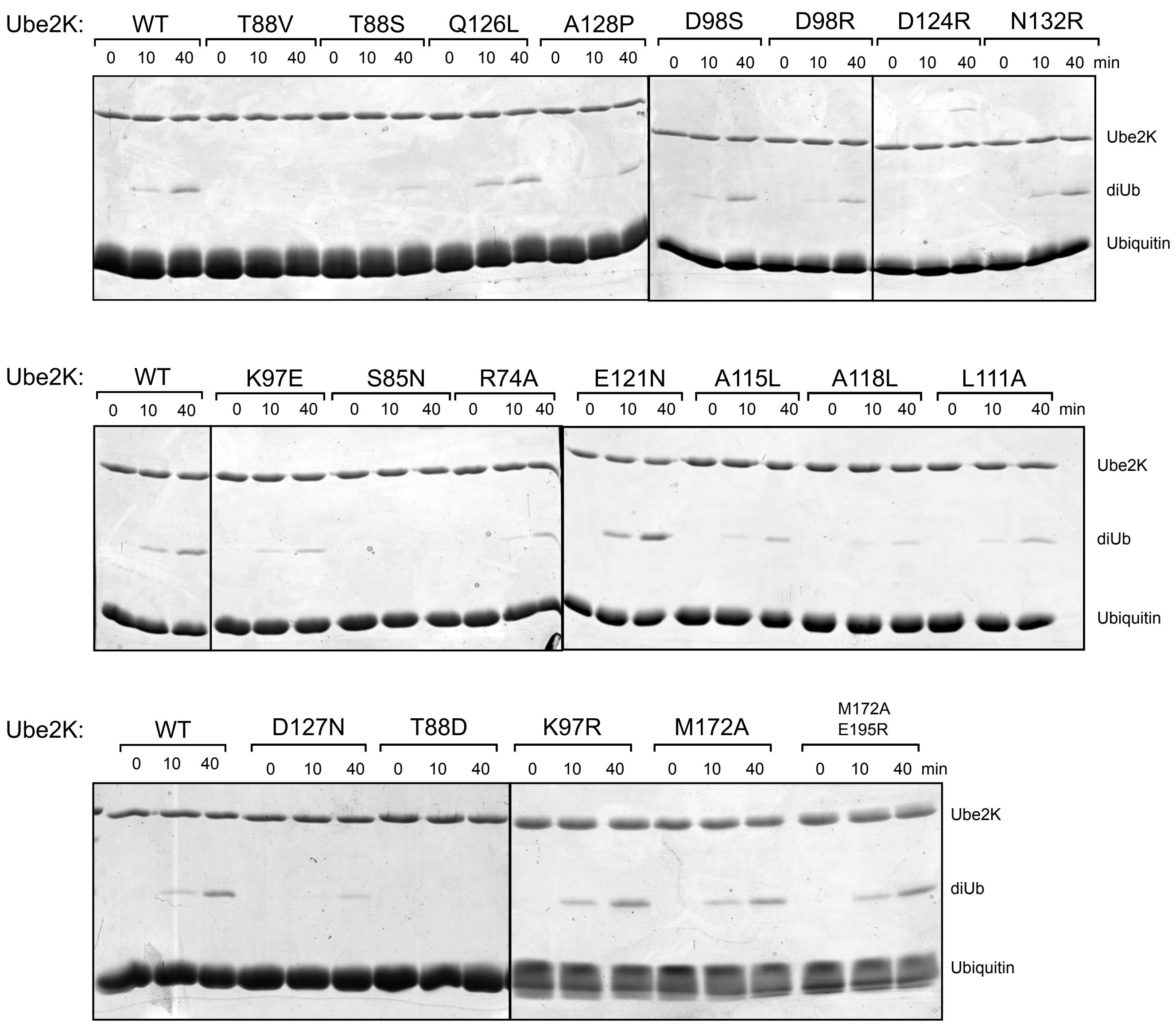

Supplementary Figure S4. Time course of initial Ube2K mutations.

Multi-turnover ubiquitylation assay showing time course of diUb formation by Ube2K and variants.

Each assay was performed in parallel with the wild-type Ube2K at the beginning of the row of gels.

Supplementary S5

a)

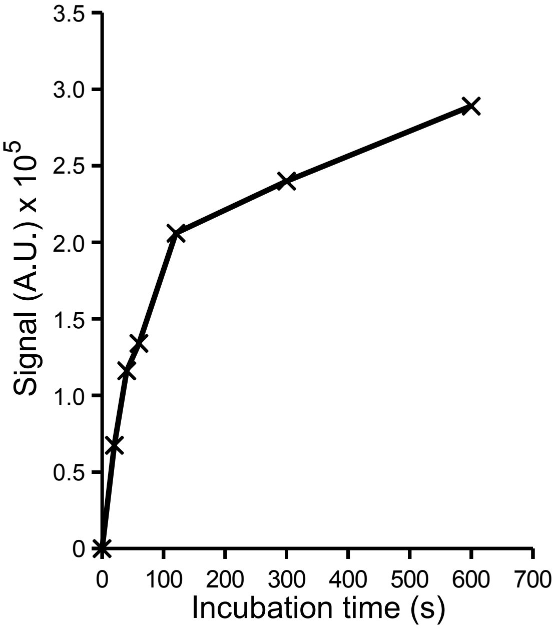

b)

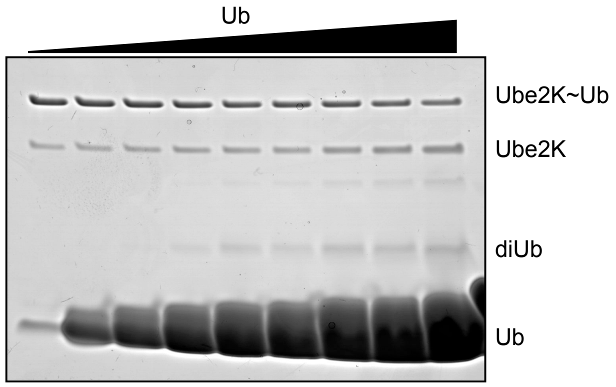

Supplementary Figure S5. DiUbiquitin kinetics experiments.

(a) Wild-type Ube2K was charged with K_0 and incubated with 1 mM wild-type ubiquitin as Ub^A. Samples were removed at the indicated times and were run on SDS-PAGE. Bands corresponding to diUb were quantified and plotted against time. This experiment was performed for all Ube2K and ubiquitin variant combinations to select a time point within the linear response of diUb formation. For wild-type Ube2K incubated with wild-type ubiquitin, 40 s was used. **(b)** Wild-type Ube2K was charged with K_0 as above and incubated with increasing amounts of wild-type ubiquitin as Ub^A. The diUb band was quantified and normalized to Ube2K~Ub and time to give apparent k_{obs} (s^{-1}).

Supplementary S6

a)

```
Ube2K     75   FITKIWHPNISSVTGAICLDILK.............DQWAAAMTLRTVLLSLQALLAAAEPDDPQDAVVAN  132
Ubc1      71   FDTKVYHPNISSVTGAICLDILK.............NAWSPVITLKSALISLQALLQSPEPNDPQDAEVAQ  128
Ube2D2    69   FTTRIYHPNIN.SNGSICLDILR.............SQWSPALTISKVLLSICSLLCDPNPDDPLVPEIAR  125
Ube2E2   123   FRTRIYHCNIN.SQGVICLDILK.............DNWSPALTISKVLLSICSLLTDCNPADPLVGSIAT  179
Ube2R1    77   FLTKMWHPNIY.ETGDVCISILHPPVDDPQSGELPSERWNPTQNVRTILLSVISLLNEPNTFSPANVDASV  146
Ube2G2    73   FTCEMFHPNIY.PDGRVCISILHAPGDDPMGYESSAERWSPVQSVEKILLSVVSMLAEPNDESGANVDASK  142
```

b)

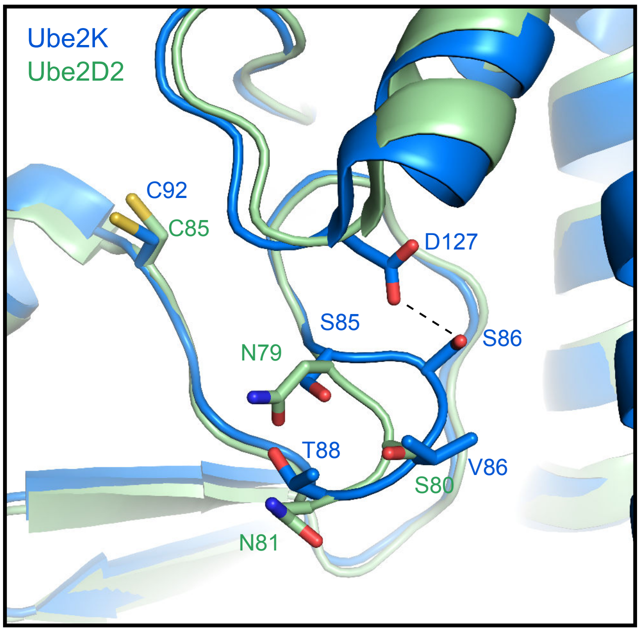

Supplementary Figure S6. Serine 86 extends the β4- α2 loop.

(a) Alignment of Ube2K and Ubc1 against other E2s. Active site Cys residues are highlighted in yellow, Ub^{A} coordinating Lys/Arg in blue, putative desolvating acidic groups in pink, and critical residues 85-88 of Ube2K in green. Identical, highly similar and similar residues are indicated by dark, medium and light grey highlights, respectively. **(b)** Overlay of Ube2K and Ube2D2 showing the extension of the β4-α2 loop in Ube2K due to the presence of Ser86. Side chains of residues 85-88 of Ube2K, 79-81 of Ube2D2 and the active site Cys are shown as sticks. Ube2K is in blue, and Ube2D2 is in green. Oxygen, nitrogen and sulphur atoms are in red, blue, and yellow, respectively.

Supplementary Table S1. DiUbiquitin synthesis kinetics assay (n=3)

Ube2K	Ubiquitin (Ub^A)	Incubation time (s)	Mean of apparent K_m (µM) (+/- Std. error)	Mean of apparent k_{cat} (s^{-1}) (+/- Std. error)
Wild type	Wild type	40	476 (+/- 196)	0.0091 (+/- 0.0017)
Wild type	E51R	300	755 (+/- 332)	0.0038 (+/- 0.00072)
Wild type	Y59L	>1200	N/D[a]	N/D[a]
K97E	Wild type	120	>3000[b]	N/D[b]
K97E	E51R	40	74.6 (+/- 18.9)	0.021 (+/- 0.0015)
Q126L	Wild type	120	>3000[b]	N/D[b]
Q126L	Y59L	600	>3000[b]	N/D[b]

[a]No significant diUb synthesis activity was observed with 2 mM Ub^A

[b]K_m was above the measurable range of the experiment, making estimation of apparent K_m and k_{cat} impossible
